# Can subjective global assessment of nutritional status predict survival in ovarian cancer?

**DOI:** 10.1186/1757-2215-1-5

**Published:** 2008-10-15

**Authors:** Digant Gupta, Carolyn A Lammersfeld, Pankaj G Vashi, Sadie L Dahlk, Christopher G Lis

**Affiliations:** 1 Cancer Treatment Centers of America^® ^(CTCA) at Midwestern Regional Medical Center, Zion, IL, USA

## Abstract

**Background:**

Malnutrition is a significant problem in patients with ovarian cancer. The goal of this study was to investigate the prognostic role of Subjective Global Assessment (SGA) in patients with ovarian cancer treated in an integrative cancer treatment setting.

**Methods:**

We evaluated a case series of 132 ovarian cancer patients treated at Cancer Treatment Centers of America^® ^from Jan 2001 to May 2006. SGA was used to assess nutritional status at baseline. Using SGA, patients were classified as well nourished (SGA A), moderately malnourished (SGA B) or severely malnourished (SGA C). Kaplan Meier method was used to calculate survival. Cox proportional hazard models were constructed to evaluate the prognostic effect of SGA independent of other factors.

**Results:**

Of 132 patients, 24 were newly diagnosed while 108 had received prior treatment. 15 had stage I disease at diagnosis, 8 stage II, 85 stage III and 17 stage IV. The median age at presentation was 54.4 years (range 25.5 – 82.5 years). 66 patients were well-nourished (SGA A), 35 moderately malnourished (SGA B) and 31 severely malnourished (SGA C). Well nourished patients had a median survival of 19.3 months (95% CI: 14.1 to 24.5), moderately malnourished 15.5 months (95% CI: 5.8 to 25.1), and severely malnourished 6.7 months (95% CI: 4.1 to 9.3); the difference being statistically significant (p = 0.0003). Multivariate Cox modeling, after adjusting for stage at diagnosis and prior treatment history found that moderately malnourished and severely malnourished status were associated with a relative risk of 2.1 (95% CI: 1.2 to 3.6, p = 0.008) and 3.4 (95% CI: 1.9 to 5.8, p < 0.001) respectively as compared to well nourished status.

**Conclusion:**

Univariate and multivariate survival analyses found that low SGA scores (i.e. well-nourished status) are associated with better survival outcomes. This study lends support to the role of aggressive nutritional intervention in improving patient outcomes in cancer care.

## Background

The overall age-adjusted incidence rate for all ovarian cancer cases as reported by the Surveillance, Epidemiology, and End Results (SEER) Program of the National Cancer Institute is 16.23 cases per 100,000 women standardized to the 2000 United States standard population [1]. Ovarian cancer is the fifth leading cause of cancer deaths in women, the leading cause of death from gynecological malignancy, and the second most commonly diagnosed gynecologic malignancy in the United States [2,3]. Most patients are diagnosed with regional and distant disease, which have poor 5-year survival rates of 69% and 29%, respectively [3].

Various clinical, biochemical and histological prognostic factors for ovarian cancer have been identified. Age, stage, grade, and cytology are important prognostic factors in high-risk early-stage epithelial ovarian cancer [4,5]. Performance status, tumor histology and residual tumor volume are independent predictors of prognosis in patients with stage III epithelial ovarian cancer [5]. Additionally, presence or absence of ascites and diameter of the largest residual tumor nodule are statistically important predictors of survival in ovarian cancer [6]. Furthermore, change of body weight during primary chemotherapy has also been reported as a strong prognostic factor [7]. Recently nutritional status has been hypothesized to be of prognostic value in patients with ovarian cancer [8].

Malnutrition in cancer patients is a significant problem due to a variety of mechanisms involving the tumor, the host response to the tumor, and anticancer therapies [9], especially among those patients diagnosed with ovarian cancer [10]. Malnutrition has been associated with a number of clinical consequences, including reduced quality of life (QoL), decreased response to treatment, increased risk of chemotherapy-induced toxicity and a reduction in survival of cancer patients [11,12] ovarian cancer being no exception [13]. The prevalence of malnutrition in patients with ovarian cancer has been reported to an extent of 67% [14,15]. As malnutrition can affect the treatment and outcomes of patients with ovarian cancer, timely intervention to assess and improve nutritional status in such patients is of utmost importance.

There are various methods of assessing nutritional status in cancer, and each has its own advantages and disadvantages. Among the most commonly used tools to measure nutritional status are anthropometric and laboratory measurements (e.g. weight change, arm muscle circumference, triceps skinfold thickness, serum albumin, transferrin assays and nitrogen balance studies) [16-21]. Anthropometric criteria alone are the most useful to assess chronic malnutrition, as alterations in body composition occur later during the malnutrition process [22]. Some of the objective measures such as serum albumin are likely to be influenced by many non-nutritional factors [23-25]. The interpretation of these measures is often difficult because non-nutritional factors, such as hydration state and disease process, can obscure the effects of actual nutrient deprivation [26]. Furthermore, some objective indicators such as serum albumin have long half-lives, thus, assessing changes in the nutritional status over a short period of time is challenging. In an effort to overcome the problems of traditional nutritional assessment, an easy-to-use, inexpensive, and non-invasive clinical instrument has been developed – the Subjective Global Assessment (SGA).

The SGA is a clinical technique that combines data from subjective and objective aspects of medical history (weight change, dietary intake change, gastrointestinal symptoms, and changes in functional capacity) and physical examination (loss of subcutaneous fat, muscle wasting, ankle or sacral edema and ascites) [27]. After evaluation, patients are categorized into three distinct classes of nutritional status; well nourished (SGA A), moderately malnourished (SGA B) and severely malnourished (SGA C). The SGA has been validated in a number of diverse patient populations, including cancer patients [28-36]. It has also been correlated with a number of objective nutritional assessment indicators, morbidity, mortality, and QoL measures [23,27,34,37-40]. To the best of our knowledge, no studies conducted to date have evaluated the prognostic significance of SGA in ovarian cancer.

The primary objective of this study is to evaluate the prognostic significance of the SGA in patients with ovarian cancer treated in an integrative cancer treatment setting

## Methods

### Study Sample

A retrospective chart review was performed on a consecutive case series of 132 ovarian cancer patients treated at Cancer Treatment Centers of America^® ^(CTCA) at Midwestern Regional Medical Center (MRMC) between January 01 and May 06. None of these patients had received any treatment at MRMC when enrolled in this investigation. The patients were identified from the MRMC tumor registry. Only patients with a histologically confirmed diagnosis of ovarian cancer were included in this study.

The SGA was used to assess nutritional status. All patients in this study were scheduled for a consultation with a dietitian. Prior to each consultation, a dietitian reviewed the patient's history from the medical record and verified the patient's current weight. During the consultation, the dietitians reviewed the SGA instrument with the patient to obtain answers to all the questions. The dietitians also completed a physical exam paying particular attention to loss of subcutaneous fat, muscle wasting, presence of ankle and sacral edema and ascites. After the consultation, the dietitians ranked the patient's nutritional status as well nourished (SGA A), moderately malnourished (SGA B) or severely malnourished (SGA C) as described by Detsky et al [27]. For the purpose of this analysis, malnutrition was defined as either SGA B or SGA C.

### Prespecified Baseline Clinical Factors

Baseline clinical factors that were assessed for prognostic significance were age at presentation, stage of disease at diagnosis and prior treatment history. The prior treatment history variable categorized patients into those who have received definitive cancer treatment elsewhere before coming to our institution and those who were newly diagnosed at our institution. The only follow-up information required was the date of death or the date of last contact/last known to be alive. This study was approved by the Institutional Review Board at Midwestern Regional Medical Center.

### Data Analysis and Statistical Methods

All data were analyzed using SPSS 11.5 (SPSS Inc., Chicago, IL, USA). Patient survival was defined as the time interval between date of first patient visit to the hospital and date of death from any cause or date of last contact/last known to be alive. The Kaplan-Meier or product-limit method was used to calculate survival. The log rank test statistic was used to evaluate the equality of survival distributions across different strata. A difference was considered to be statistically significant if the p value was less than or equal to 0.05. Survival was also evaluated using univariate and multivariate Cox regression analysis. Variables evaluated included SGA, age at presentation, prior treatment history, and stage at diagnosis. For the purpose of this analysis, stage at diagnosis variable was treated as a dichotomous variable with 2 categories – early stage (stages I and II) and late stage (stages III and IV).

## Results

At the time of this analysis (June 08), 91 patients had expired and 41 were censored, as shown in Table 1. The cut-off date for the follow-up for all participants was June 08. The median age at presentation was 54.4 years (range 25.5 – 82.5 years). 66 patients were well nourished (SGA A), 35 were moderately malnourished (SGA B) and 31 were severely malnourished (SGA C). Of 24 analytic patients, 9 (37.5%) were well-nourished while 57 (52.8%) of 108 non-analytic patients were well-nourished, the difference being statistically non-significant (p = 0.32). Of 23 early-stage (stage I and II) patients, 13 (56.5%) were well-nourished while 51 (50.0%) of 102 late-stage (stage III and IV) patients were well-nourished, the difference being statistically non-significant (p = 0.20).

**Table 1:** Patient Characteristics

***Characteristic***	***Categories***	***Number***	***Percent (%)***
Vital Status	Expired	91	68.9
	Censored^1^	41	31.1
Prior Treatment History	Progressive disease	108	81.8
	Newly diagnosed	24	18.2
Stage at Diagnosis	Stage I	15	11.4
	Stage II	8	6.1
	Stage III	85	64.4
	Stage IV	17	12.9
	Missing	7	5.3
Age at Presentation	Mean	53.4	
	Median	54.4	
	Range	25.5 – 82.5	
SGA	A	66	50
	B	35	26.5
	C	31	23.5

^1^Patients who reached the end of their follow-up without experiencing death.

N = 132

Table 2 shows the univariate survival analysis of different prognostic factors. SGA and treatment history were found to be statistically significantly associated with survival. Stage at diagnosis was found to be marginally significant and it was decided to control for it in the multivariate analysis. Age at presentation and BMI were not found to be statistically significantly associated with survival and were therefore not considered further.

**Table 2:** Univariate Kaplan-Meier Survival Analysis

***Variable***	***Median survival in months***	***Log-rank score***	***P-value***
SGA			
• Well nourished	19.3 (14.1 to 24.5)	15.9	0.0003
• Moderately malnourished	15.5 (5.8 to 25.1)		
• Severely malnourished	6.7 (4.1 to 9.3)		
Tumor Stage			
• Stage I and II	23.9 (7.7 to 40.3)	3.3	0.07
• Stage III and IV	15.5 (10.0 to 20.9)		
Treatment History			
• Newly diagnosed	43.1 (18.1 to 68.1)	13.5	0.0002
• Progressive disease	12.1 (7.2 to 17.1)		

N = 132

**Figure 1:**
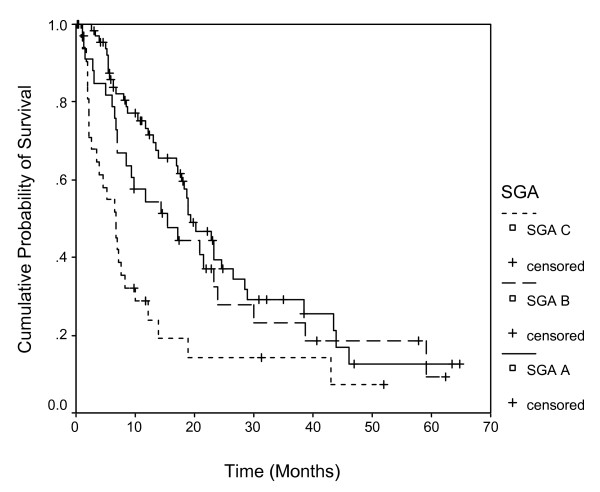
**Survival stratified by 3 categories of SGA.** Each drop in a probability curve indicates one or more events in that group. Vertical lines indicate censored patients, i.e., those who reached the end of their follow-up without experiencing death.

Figure 1 shows the survival curves for the 3 categories of SGA. Well nourished patients had a median survival of 19.3 months (95% CI: 14.1 to 24.5), moderately malnourished 15.5 months (95% CI: 5.8 to 25.1), and severely malnourished 6.7 months (95% CI: 4.1 to 9.3); the difference being statistically significant (p = 0.0003).

Table 3 summarizes the results of multivariate Cox regression analyses. Multivariate Cox modeling, after adjusting for stage at diagnosis and prior treatment history found that moderately malnourished status was associated with a relative risk of 2.1 (95% CI: 1.2 to 3.6, p = 0.008) as compared to well nourished status. Similarly, severely malnourished status was associated with a relative risk of 3.4 (95% CI: 1.9 to 5.8, p < 0.001) as compared to well nourished status. Prior treatment history and stage at diagnosis were also found to be statistically significantly associated with survival independent of SGA as shown in Table 3. It was interesting to see that stage at diagnosis which was marginally significant upon univariate analysis became statistically significant upon multivariate analysis.

**Table 3:** Multivariate Cox Proportional Hazard Model

Independent Variable	Unit of increase	RR^1^	95% CI	P-value
Moderately malnourished	Well-nourished as referent	2.1	1.2, 3.6	0.008
Severely malnourished	Well-nourished as referent	3.4	1.9, 5.8	< 0.001
Stage at Diagnosis	Stage I and II as referent	2.1	1.1, 4.0	0.02
Treatment History	Newly Diagnosed as referent	4.8	2.4, 9.7	< 0.001

^1^Relative risk (Cox proportional hazard)

N = 132

Table 4 shows statistically distinct prognostic classes of our patient cohort. Stratum 1 has no median survival because all 3 observations were censored.

**Table 4:** Prognostic Classes of Ovarian Cancer Patients

***No***.	***Strata***	***N***	***Median Survival (in months)***	***95% CI***
1	Well nourished, stage I and II, newly diagnosed	3	-	-
2	Well nourished, stage III and IV, newly diagnosed	6	43.5	13.3 to 73.8
3	Well nourished, stage I and II, progressive disease	10	18.7	10.0 to 27.4
4	Well nourished, stage III and IV, progressive disease	45	18.9	16.9 to 21.0
5	Malnourished, stage I and II, progressive disease	10	23.9	5.9 to 42.1
6	Malnourished, stage III and IV, progressive disease	36	6.7	5.9 to 7.6

N = 132

## Discussion

The identification of prognostic factors in ovarian cancer is of considerable importance for the clinical management of the disease. While nutritional status has been hypothesized to have an association with survival, the published literature documenting its prognostic significance in ovarian cancer remains sparse. Despite the number of nutrition assessment tools used for research purposes, a consensus has not been reached on what may be the "gold standard" for nutritional assessment in cancer. The current study was undertaken to investigate if SGA, a potential indicator of nutritional status, could predict survival in ovarian cancer.

In this study, we found that SGA A (well-nourished status) versus SGA B/C (moderate to severe malnourished status) identified patients with better survival outcomes. We found that the SGA provides useful prognostic information in patients with ovarian cancer. In a clinical setting, the SGA is invaluable in identifying malnourished patients in a quick and non-invasive manner. Moreover, the simplicity of use of the SGA also enables health professionals other than oncologists and dietitians to accurately assess the patient's nutritional status. In our previous study conducted in colorectal cancer, we found SGA to be a significant predictor of survival. The median survival of patients with SGA A was 12.8 months (95% CI; 9.1–16.5), those with SGA B was 8.8 months (95% CI; 6.7–10.9) and those with SGA C was 6 months (95% CI; 3.9–8.1) [41].

SGA is simple, safe and inexpensive, which renders it a universal tool for nutritional assessment. SGA differs from other nutritional assessment methods in that it is the only one that evaluates functional capacity [42]. SGA has gained acceptance among investigators and it is now used as a benchmark to validate new assessment methods, such as bioelectrical impedance analysis [43] and mid-upper arm anthropometry. One of the major criticisms of the method is that its accuracy depends on the observer's experience. Although SGA depends on the interviewer's training and on the interpretation of the collected data, its subjectivity may be minimized by assigning points to questionnaire items [36]. Another criticism directed at SGA is that it is a subjective method with only three categories, which does not allow assessment of nutritional scale on a continuum [42]. Despite these disadvantages, SGA continues to be a good option for assessing nutritional status in several clinical conditions.

This study, because of its retrospective nature, relies on data not primarily meant for research. We think that restricting the analysis to newly diagnosed patients (patients with no prior treatment history) would have been more accurate, since it would have allowed for evaluation of true overall survival time i.e. time from the date of diagnosis to the date of death. However, doing so would have caused a significant reduction in the sample size. In our study, the survival time was calculated from the day of first visit at our hospital because information on SGA was not available at the time of diagnosis for previously treated patients. This drawback emphasizes the need for conducting prospective studies having nutritional information available since the date of diagnosis. A majority of our patients had advanced stage disease and had failed primary treatment elsewhere before coming to our hospital. As a result, generalizability of the study findings to cancer patients with early-stage disease might be questionable. However, we have no reasons to believe that patients with early-stage disease will display different findings. This study did not evaluate the effectiveness of nutritional intervention on survival and future prospective studies should attempt to address this important research question. The SGA, being a subjective method, relies on the observer's ability to collect and interpret information, and as a result, is likely to suffer from observer bias. No assessment of inter-rater reliability of the users of the SGA was made in this study. This bias, however, was minimized by restricting the use of the SGA to well-trained dietitians with an expertise in the use of this clinical instrument.

## Conclusion

In summary, our study has demonstrated the prognostic significance of SGA in ovarian cancer. To the best of our knowledge, this is the first study to evaluate SGA for its prognostic importance in ovarian cancer patients treated in an integrative cancer treatment setting.

## Competing interests

The authors declare that they have no competing interests.

## Authors' contributions

DG, CAL, and SLD participated in concept, design, data collection, data analysis, data interpretation and writing. PGV participated in concept, design and data interpretation. CGL participated in concept, design, data interpretation and general oversight of the study. All authors read and approved the final manuscript.
